# A global bibliometric and visualized analysis of bacterial biofilm eradication from 2012 to 2022

**DOI:** 10.3389/fmicb.2023.1287964

**Published:** 2023-11-22

**Authors:** Tao Wang, Rui Zhang, Zhiling Chen, Peipei Cao, Qionglin Zhou, Qiang Wu

**Affiliations:** ^1^The First Affiliated Hospital, Hainan Medical University, Haikou, China; ^2^International School of Public Health and One Health, Hainan Medical University, Haikou, China; ^3^The Second Affiliated Hospital, School of Tropical Medicine, Hainan Medical University, Haikou, China; ^4^School of Basic Medical Sciences and Life Sciences, Hainan Medical University, Haikou, China

**Keywords:** bacteria biofilm eradication, bibliometric and visualized analysis, dynamic trends, focal points, emerging topics

## Abstract

**Background:**

To deeply explore the dynamic trends, focal points and emerging topics of bacterial biofilm eradication field and provide novel insights for prospective research endeavors, the first global bibliometric and visualized analysis of the field was employed in this study.

**Methods:**

The study meticulously curated articles and reviews concentrating on biofilm eradication from the Web of Science Core Collection (WoSCC) and identified literature published in 2012–2022 for further analysis, and the bibliometric and visualized analysis was performed to elucidate a clustering pattern in the domain with tools mainly including CiteSpace and VOSviewer.

**Results:**

15,503 authors affiliated with 2,397 institutions spanning 96 countries or regions contributed to a corpus of 3,201 articles, containing 7,005 keywords. The USA emerged as a commanding vanguard in exploring the antibiofilm strategies and displaying pioneering initiatives within this sphere. The Chinese Academy of Sciences (CAS) emerged as the most prolific source of publications. Noteworthy among authors, Pandian Shunmugiah Karutha secured the lead in article contributions as well as co-citations while Deng Le with his team is poised to become a dominant influence in the future. Despite that, the extent of collaborative engagement across different institutions and authors appeared to fall short of its potential. Frontiers in Microbiology led the discourse by publishing a substantial body of articles and standing as the most recurrently co-cited publication. The most influential research domains encompassed “bacterial biofilm formation, “photodynamic therapy” and “phage therapy.” Recent trends and forefronts concentrate on intensifying research into facilitating the shift of multiple strategies for biofilm eradication from controlled lab settings or animal studies to real-world clinical environments.

**Conclusion:**

Fundamentally, this study presents a comprehensive scrutiny and reveals that the realm of bacterial biofilm eradication is undergoing rapid evolution, with even greater expansion anticipated in the times ahead. Subsequent scholars should emphasize the augmentation of collaborative efforts and focus their energies on emerging topics, thus contributing to break through current barriers in transitioning biofilm eradication strategies from the “fundamental” stage to “practical” application.

## 1 Introduction

### 1.1 Research background

Bacterial biofilms are intricate structures comprised of bacteria and other microorganisms, which could be found on various substrates such as rocks in water, the inner linings of pipes, medical devices, and human tissues, contributing to chronic bacterial infections that affect both humans and other organisms as well as posing prominent health concerns worldwide due to their ability to develop multidrug resistance, evade host defenses, and withstand various stresses (Subhadra et al., 2018; Sharma et al., 2019). A bacterial biofilm is characterized by the formation of complex three-dimensional communities, consisting of diverse bacterial colonies tightly embedded in an extracellular polymeric substance (EPS) matrix (Kostakioti et al., 2013). In addition to their impact on health, biofilms also play a destructive role in industrial settings, such as causing persistent pollution, pipeline blockage and corrosion (Lenhart et al., 2014). Consequently, research focused on biofilm eradication holds paramount clinical and practical significance.

### 1.2 Literature review and research gap

For over a century, bacteria have been the subject of extensive laboratory investigation. However, it was a simple observation in 1943 that triggered a profound shift in our understanding: bacteria exhibit a preference for communal living (Zobell, 1943). Afterwards, it wasn’t until 1982 that researchers, while examining bacteria firmly adhered to a pacemaker lead retrieved from a patient with recurrent bacteremia, embarked on the inaugural study of biofilms (Marrie et al., 1982). This milestone report also represented one of the earliest references to “biofilm-forming bacteria” in medical literature, sparking heightened interest in biofilm-related infections. Over the past 40 years, microbiologists have broadly acknowledged that bacteria in their natural habitat manifest in two distinct life forms: in one scenario, they exist as solitary, independent, and free-floating cells (planktonic); in the other, bacteria coalesce into microbial aggregates known as biofilms. Moreover, the term “biofilm” has evolved from its original reference to surface-associated biomaterials to specifically signify clustered bacteria communities (Costerton et al., 1978; McCoy et al., 1981).

Early biofilm research was predominantly centered on engineering applications and the descriptive characterization of biofilms. However, with the revelation of specific gene regulation governing surface attachment *in vitro*, and the introduction of *in vitro* systems in the laboratory to investigate biofilm formation and phenotypes, the research focus transitioned toward delving into the fundamental molecular mechanisms of biofilm formation. This encompasses the role of cellular signaling in collective genetic control (Davies et al., 1998), as well as the utilization of genetic tools to pinpoint the genes essential for surface adhesion and subsequent biofilm development (O'Toole and Kolter, 1998; Schembri et al., 2003). The application of molecular genetics further advanced the advent of novel technologies for scrutinizing biofilm communities, yielding fresh insights into the molecular genetic underpinnings of biofilm maturation (Davey and O'Toole, 2000). In recent decades, the emergence of biofilm formation has presented substantial challenges to human beings, as a result, the advent of novel approaches for combating and forestalling biofilm development has garnered significant interest, and the implementation of tailored control measures to impede biofilm formation has evolved into a swiftly progressing domain (Srinivasan et al., 2021; Abdelhamid and Yousef, 2023). In brief, scientists have made significant advancements in employing a range of physical, chemical, and biological approaches for eliminating biofilms. Based on earlier reports, antibiotics demonstrate heightened efficacy when targeting biofilms in their early developmental stages, as opposed to mature biofilms, consequently, a preference for combination therapy over solitary antibiotic treatment has emerged (Herrmann et al., 2010). Research underscores the potential of anti-fouling or antimicrobial surfaces as a plausible alternative for impeding biofilm formation (Francolini et al., 2014). Notably, hydrophilic polymer coatings are harnessed to engineer anti-fouling surfaces, exerting substantial reductions in microbial adhesion. Furthermore, coatings infused with nanoparticles, such as silver nanoparticles, and antioxidative nanoparticles, present additional avenues for curtailing biofilm formation (Crisante et al., 2016). Photodynamic therapy (PDT) harbors promising implications for averting biofilm infections in wounds, employing photosensitive dyes that, upon illumination in the presence of oxygen, effectively exterminate bacteria (Darmani et al., 2018; Gholibegloo et al., 2018). Another burgeoning approach involves the utilization of potent anti-biofilm molecules or agents (enzymes, peptides, antibiotics, polyphenols, and more) capable of dissolving existing biofilms by disrupting bacterial signaling pathways (Donelli et al., 2007; Daboor et al., 2019; Scheper et al., 2021). Moreover, bacteriophages, viruses that specifically infect and destroy bacteria, have also been identified as an effective strategy for biofilm eradication (Gutiérrez et al., 2016; Kortright et al., 2019; Ferriol-González and Domingo-Calap, 2020). Despite extensive endeavors and a multitude of innovative approaches by researchers at the laboratory level in combating biofilms, there remains a notable scarcity of practical applications, particularly in clinical settings (Mohamad et al., 2023). The formidable divide between research and application underscores a significant shortfall in current studies. Thus, the urgent task at hand is to empower researchers in the realm of biofilm formation mechanisms and diverse anti-biofilm strategies to surmount their individual blind spots, unearth additional avenues for collaboration, and pinpoint cross-disciplinary fusion points.

### 1.3 The main work and innovations

At present, while there have been teams report reviews in the domain of biofilm eradication, it is vital to acknowledge that these reports primarily highlight specific advancements in certain directions, and other pertinent aspects of the research are not addressed. Consequently, there arises a need to employ specialized tools for a comprehensive analysis of both the external and internal attributes of the literature generated in this field. This approach will furnish researchers with multi-faceted guidance and evaluative insights. Bibliometrics is a widely employed way that enables qualitative and quantitative analysis of impactful publications within a specific subject area, allowing for the examination of extensive volumes of publications and their production trends, both at macroscopic and microscopic scales, proving valuable in scrutinizing the historical context of research literature generation (Kokol et al., 2021). It combines systematic statistical methods and sophisticate mathematical devices with visualized data techniques to uncover the knowledge structure, dynamic trends, focal points, and emerging topics in a specially appointed field. By utilizing bibliometrics, it becomes possible to identify posting trends, influential authors, institutions, countries, and publications (Gao et al., 2019; Hu et al., 2021). Furthermore, managing a bibliometric analysis of existing exploration allows for the objective assessment of landmark literature and peer recognition by examining highly cited publications within a particular field (Suero-Abreu et al., 2021).

Advancements in the study of bacterial biofilms offer fresh perspectives into their formation and resistance mechanisms while serving as a foundation for the development of new biofilm eradication methods. However, the complexity and diversity of bacterial biofilms continue to present challenges in investigation. Fueled by advancements in biomedical engineering and a greater comprehension of the complicated process of biofilm formation, the study of the given field has garnered great interest, resulting in a substantial number of articles published in the past decade especially after the year 2012 (Chen et al., 2016; de Breij et al., 2018). Considering this, we have implemented the first bibliometric analysis of publications on this selected topic to offer valuable insights into the present status and future research directions with tools mainly including CiteSpace and VOSviewer due to these softwires has found extensive application in bibliometric analysis and is widely acknowledged in network and visualization analysis (Cheng et al., 2022; Wang and Maniruzzaman, 2022; Xu et al., 2022). Moreover, our search strategy did not retrieve valid data prior to 2012 and papers published in 2023 are still in a state of dynamic updating, so we identified literature published in 2012–2022 for our analysis. Our aim is going to serve as a precious resource for researchers seeking to delve deeper into this promising research domain by addressing the following questions:

Q1: Based on the information gleaned from published literature, what is the current global dynamic trends of the field?

Q2: In this field, which countries/regions, institutions, journals, and authors have demonstrated the highest levels of productivity and influence?

Q3: What are the primary areas of research focal points and the emerging topics of intense interest for upcoming research?

## 2 Materials and methods

### 2.1 Bibliometric data source

**Figure 1:**
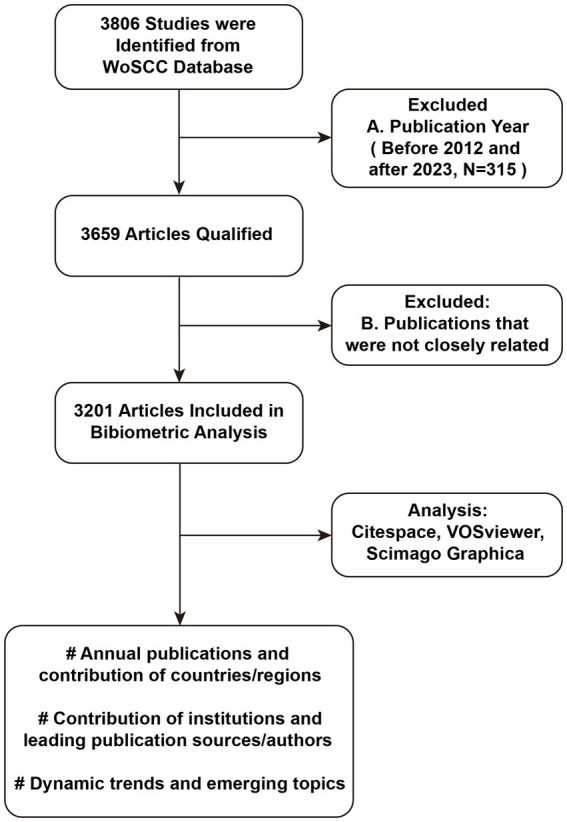
Workflow diagram of the literature search and screening of articles about bacterial biofilm eradication.

Considering the importance of obtaining high-quality and accurate results for analysis, bibliometric data was sourced from the WoSCC database on 1 April 2023 and screened out articles published from 2012 to 2022. The retrieval terms used for the title and author keywords were (“bacteri*” OR “bacill*”) AND (“biofilm*”) AND (“eradicat*” OR “anti-biofilm*” OR “antibiofilm*” OR “anti-bacteri*” OR “antibacteri*” OR “anti-microbial” OR “antimicrobial” OR “repress*” OR “suppress*” OR “clear*” OR “treat*” OR “therap*” OR “control*” OR “inhibit*” OR “combat*” OR “prevent*” OR “fight*” OR “interfer*” OR “eliminat*” OR “regulat*” OR “monitor*” OR “clean*” OR “remov*”). A total of 3,659 records were initially retrieved, further refinement eliminated publications that were not closely related, resulting in a final collection of 3,201 publications. Within this dataset, there were 7,005 keywords, 15,503 authors from 2,397 institutions, and representation from 96 countries/regions. The workflow diagram of the literature search and screening of articles about bacterial biofilm eradication was shown in Figure 1.

### 2.2 Bibliometric analysis

Firstly, we utilized the “Analyze search results” function of Web of Science to organize the annual number, disciplines, and sources of publications. Next, we exported the basic information of 3,201 search records into Microsoft Excel 2019. Subsequently, we employed VOSviewer 1.6.18, Citespace 6.1.R6, and Scimago Graphica Beta 1.0.34 for bibliometric and knowledge mapping analysis. VOSviewer portrays the interconnections and offers diverse visualization options for the scientific landscape, including illustrate associations between terms, grouping related terms, identifying co-occurring author keywords, and visualizing bibliometric or citation networks (van Eck and Waltman, 2010; Kokol et al., 2018). CiteSpace empowers scientists to gain an understanding of the intellectual structure and progression of a research domain by visually illustrating the connections in basic characteristics of literature (Synnestvedt et al., 2005). Scimago Graphica is a visualization tool that enables users to explore and analyze scientific data in a visual manner^1^. This analysis encompassed the number of papers published, countries, institutions, funding sources, authors, subject categories, disciplines, and journals. Additionally, we consulted established analytical methods and employed bibliometric mapping analysis to elucidate a clustering pattern within the realm of biofilm eradication (Kokol et al., 2022). In shortly, we scrutinized the interconnections among nodes within each cluster, segmenting them into distinct sub-categories. Then, we conducted a thorough examination of these sub-categories, affixing thematic labels to the clusters and pinpointing research dimensions. Ultimately, we conducted a comprehensive cross-analysis of themes and research dimensions to pinpoint correlated concepts, so that to exploring the primary research topics and development trends.

## 3 Results

### 3.1 Overview of annual publications and contribution of countries/regions

**Figure 2:**
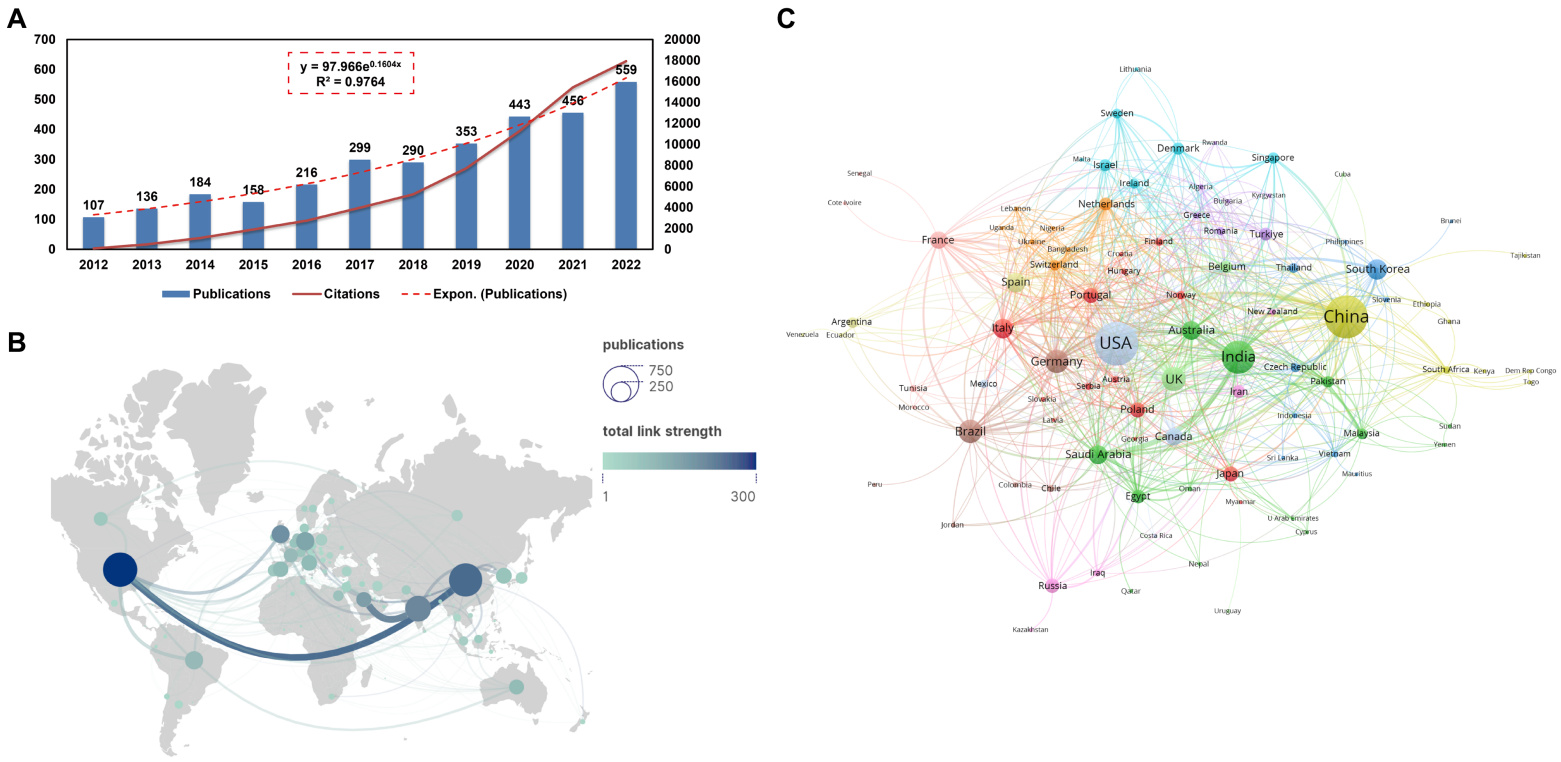
**(A)** Publications’ annual count over the past 11 years, along with a fitted curve depicting the overall growth trend (*R*^2^ = 0.9764) and the annual cumulative citation curve. **(B)** A visual network map illustrating collaborations among countries/regions, created using Scimago and VOSviewer. Node sizes reflect publication quantities, with larger nodes denoting higher counts. Line thickness indicates collaboration strength between pairs of countries/regions. Nodes transition in color from green to blue, indicating increasing article numbers. **(C)** VOSviewer’s analysis of country co-authorships. Each country is portrayed as a node, and links between countries denote co-authorship affiliations. Node sizes correspond to total publication numbers.

The volume of academic publications can serve as an indicator of the scale and growth dynamics within a specific study field. Figure 2A illustrates a consistent upward trend in the annual publication volume from 2012 to 2022. The increasing pattern in annual issuance can be described by the equation *y* = 97.966e^0.1604X^ with an *R*^2^ value of 0.9764, where y represents the annual publications and X denotes years since 2012. Table 1 presents Top 15 productive countries/regions related to bacterial biofilm eradication. The USA accounted for 19.56% (626/3201) of the total publications, followed by China with 18.65% (597/3201), India with 11.09% (355/3201), UK with 5.84% (187/3201), Germany with 5.65% (181/3201), Brazil with 5.47% (175/3201), South Korea with 4.19% (134/3201), Italy with 4.12% (132/3201), and Spain with 3.94% (126/3201). In total, research papers on this topic were published by 96 countries or regions. However, when considering the total number of citations, the USA articles received 18,620 citations, while China accumulated 12,446 citations, resulting in an average of approximately 29.74 citations per article. The H-index, which measures the impact and productivity of articles, was 68 for the USA (ranked first), 52 for China (ranked second), and 47 for India (ranked third).

**Table 1:** Top 15 productive countries/regions related to bacterial biofilm eradication.

Ranking	Country	NP	NC	TLS	AC	H-index
1	USA	626	18,620	300	29.74	68
2	China	597	12,446	250	21.17	52
3	India	355	8,873	203	24.99	47
4	UK	187	4,143	190	24.09	37
5	Germany	181	4,528	147	25.02	36
6	Brazil	175	3,480	96	19.89	33
7	South Korea	134	2,458	75	18.34	27
8	Italy	132	4,253	99	32.22	35
9	Spain	126	4,557	99	36.17	37
10	Saudi Arabia	121	2,623	161	21.68	27
11	Australia	118	4,344	94	36.81	35
12	France	104	3,587	95	34.49	32
13	Canada	102	4,081	65	40.01	31
14	Portugal	79	2,423	58	30.67	27
15	Japan	73	1,042	28	14.27	15

Ranking: according to the number of total publications. NP, total number of publications; NC, total number of citations; TLS, total link strength; AC, average citations per item.

The national collaborations map (Figure 2B) provides a visual representation of these collaborations. Most articles are contributed by authors from Asia, the Americas, and Europe. China, the USA, and India are highlighted in dark blue color, indicating a stronger link with other countries. Saudi Arabia with India (52) as well as China with the USA (51) exhibit wider linkage widths, suggesting their active engagement in collaborations. Further analysis reveals that the total link strength of the USA (300) is larger than that of China (250). Some countries have not established close collaborations with others, such as Estonia (0) and Kuwait (0), which are not represented in the graph. The VOSviewer analysis of country cooperation includes a minimum of 10 papers from 47 countries/regions. The USA, China, and India are observed to be central in this network, as evident from the overlay visualization (Figure 2C).

### 3.2 Contribution of institutions and leading publication sources

**Figure 3:**
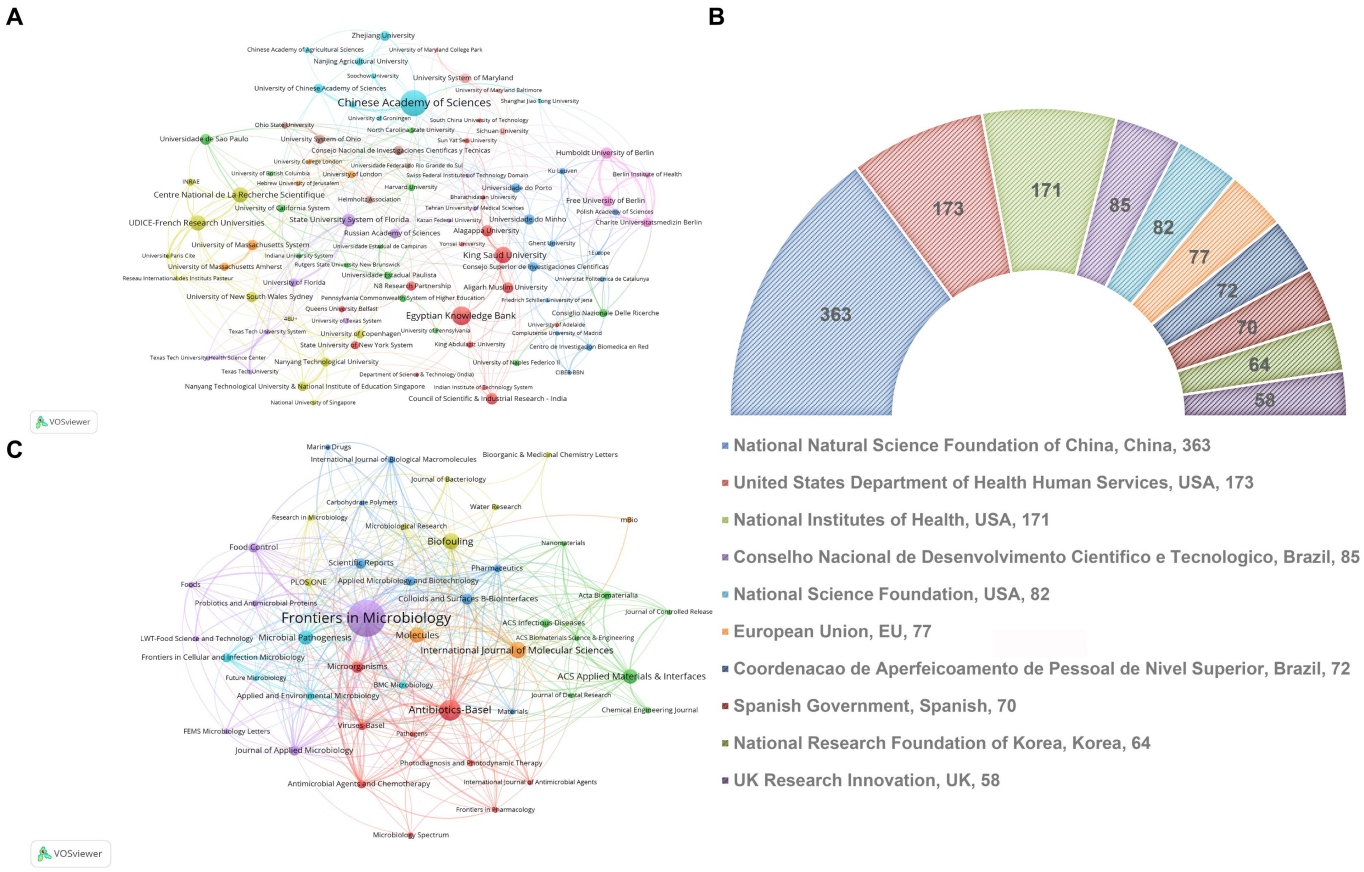
**(A)** A visual network map showcasing co-authorship institutions, where node sizes correspond to publication quantities. **(B)** The leading ten funding departments within the realm of bacterial biofilm eradication. **(C)** An overlay visualization map depicting co-occurrence analysis among journals. Nodes within this map are color-coded to indicate distinct journal clusters. Node sizes reflect co-occurrence frequency, and connections between nodes denote relationships among co-occurring journals.

As shown in Figure 3A and Table 2, the CAS, a renowned academic institution for natural sciences in China, exhibits the highest contribution in terms of publications (81), H-index (28), and total citations (2600). The second one is attributed to the Egyptian Knowledge Bank, but has no high average citations per article, total citations, or H-index. Regarding average citations per article, CAS has 32.1 citations per article, while the top-ranking institution, Aligarh Muslim University in India, has 44.94 citations per article. Figure 3A illustrates 89 institutions with at least 14 publications, the institutions displaying the greatest count of publications encompass the CAS, the Egyptian Knowledge Bank, King Saud University, UDICE French Research Universities, and Centre National de La Recherche Scientifique (CNRS). Additionally, we have assembled a list of the foremost 10 funding agencies that exhibit substantial engagement in the domain, wherein over 50% of the funding origins can be traced back to China and the USA (Figure 3B).

**Table 2:** Top 10 most active institutions of publications in bacterial biofilm eradication field.

Ranking	Institutions	NP	Citation	AC	H-index
1	Chinese Academy of Sciences	81	2,600	32.10	28
2	Egyptian Knowledge Bank	58	968	16.69	19
3	King Saud University	50	1,521	30.38	19
4	UDICE French Research Universities	48	2079	43.31	22
5	Centre National De La Recherche Scientifique	47	1,495	31.81	21
6	State University System of Florida	41	658	16.05	17
7	Council of Scientific Industrial Research India	36	805	22.36	16
8	Humboldt University of Berlin	36	926	25.72	19
9	Alagappa University	35	1,348	38.51	19
10	Aligarh Muslim University	34	1,528	44.94	18

Ranking: according to the number of total publications. NP, total number of publications; NC, total number of citations; AC, average citations per item.

As shown in Table 3, from 2012 to 2022, the top 10 major journals contributed a total of 546 articles on bacterial biofilm eradication. Among them, “Frontiers in Microbiology” had the highest number of publications (147), with 5,220 citations, securing the top rank in both categories. “Antibiotics-Basel” ranked second with 70 publications, closely followed by “Biofouling” (51) and “International Journal of Molecular Sciences” (51). In terms of citation frequency, “ACS Applied Materials & Interfaces” ranked second with 1,549 citations despite having only 45 publications. The third most cited journal was “Microbial Pathogenesis” with 1,364 citations. This was followed closely by “Biofouling” (1,285 citations) and “Molecules” (1,267 citations). As depicted in Figure 3C, the VOSviewer software was used to visualize plots of journals with at least 12 publications, resulting in a selection of 47 journals. In terms of total link strength, “Frontiers in Microbiology” ranked first with 326, followed by “Antibiotics-Basel” (142) and “International Journal of Molecular Sciences” (79), and it is evident from the graph that these journals are currently the most influential ones in the field.

**Table 3:** Top 10 most productive journals in bacterial biofilm eradication field.

Rank	Journal	ISSN	Country	IF-2022*	NP	NC	H-index
1	Frontiers in Microbiology	1,664–302X	Switzerland	5.2	147	5,220	40
2	Antibiotics Basel	2079–6,382	Switzerland	4.8	70	1,257	21
3	Biofouling	0892–7,014	England	2.7	51	1,285	25.2
4	International Journal of Molecular Sciences	1,422–0067	Switzerland	5.6	51	808	15.84
5	Molecules	1,420–3,049	Switzerland	4.6	46	1,267	27.54
6	ACS Applied Materials Interfaces	1944–8,244	USA	9.5	45	1,549	34.42
7	Microbial Pathogenesis	0882–4,010	England	3.8	42	1,364	32.48
8	Microorganisms	2076–2,607	Switzerland	4.5	33	597	18.09
9	Colloids and Surfaces B-Biointerfaces	0927–7,765	Netherlands	5.8	32	1,033	32.28
10	Scientific Reports	2045–2,322	Germany	4.6	29	796	27.45

Ranking: according to the number of total publications. *NP, total number of publications; IF, impact factor; NC, total number of citations.

### 3.3 Authors, co-cited authors, and subject categories

**Figure 4:**
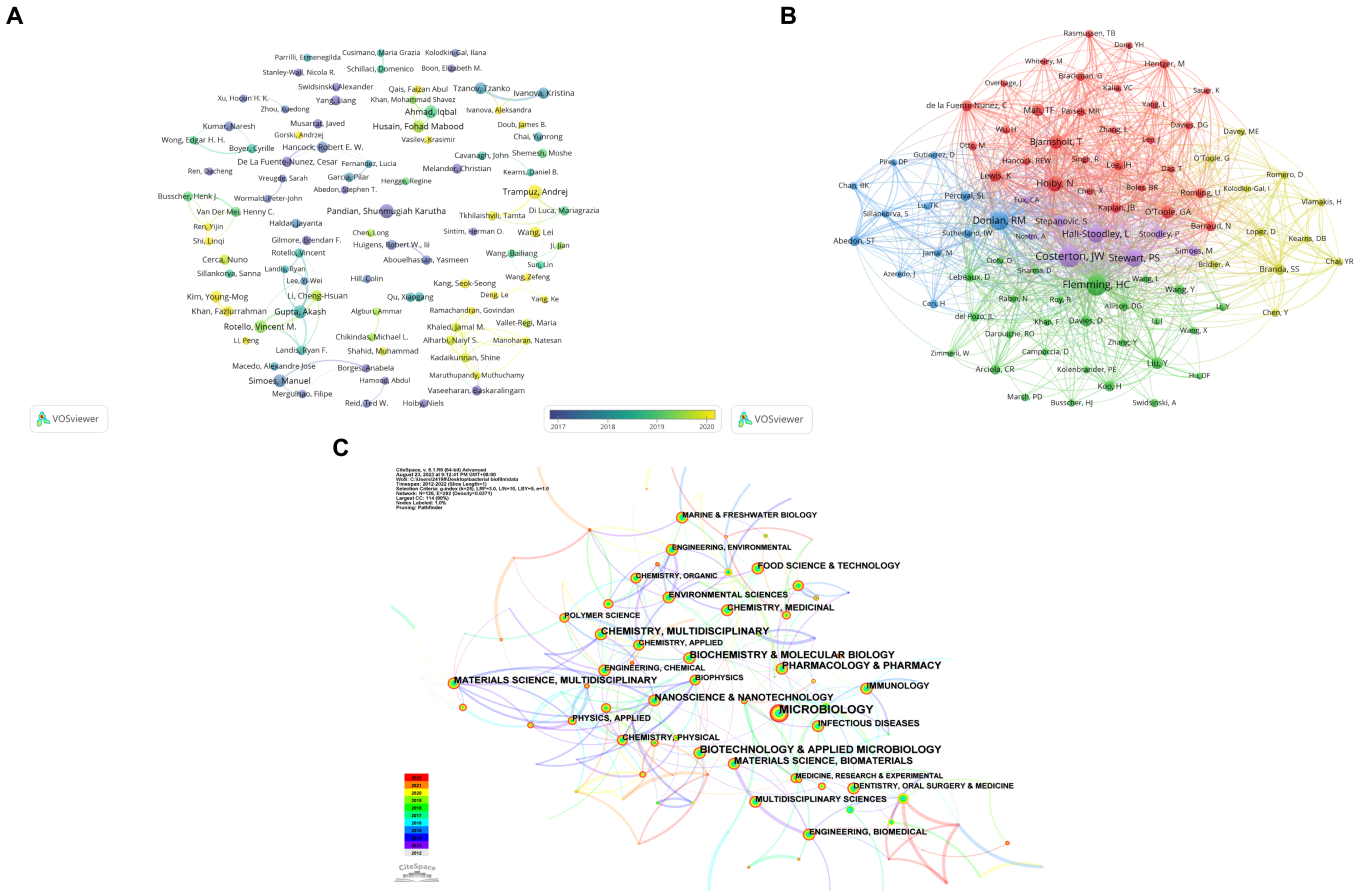
**(A)** A visual network map depicting the analysis of author co-occurrence, highlighting connections between authors who have collaborated closely. The color spectrum, spanning from purple to yellow, signifies the temporal proximity of publications to either 2016 or 2021. **(B)** An analysis of author co-citation, with nodes representing individual authors and lines symbolizing co-citation relationships. **(C)** A network visualization of co-occurring subject categories related to bacterial biofilm eradication, generated using CiteSpace.

Author co-authorship analysis is depicted in Figure 4A, and the top 10 most productive journals is shown in Table 4, the graph displays scholars who have published at least 6 papers, showcasing a relatively loose collaboration among them. The main research teams are formed at different times based on the average year of publication. Noteworthy scholars in this network include Gupta Akash from University of Massachusetts, possessing the highest total link strength of 44 and an average publication year of 2018. Rotello Vincent M from University of Massachusetts follows closely with the second total link strength of 30 and an average publication year of 2019. This collaborative team consists of 7 members, with the weakest total linkage observed between Rotello Vincent and Landis Ryan, both with a total linkage strength of 17. Pandian Shunmugiah Karutha, affiliated with Alagappa University, distinguishes themselves by achieving the highest publication count of 18, an average publication year of 2014, and the most substantial total citations at 959, indicating their status as an early and influential researcher in this field. Deng Le from Hunan Normal University and his team members, on average, have a publication year after 2021, with Yang Ke from Hunan Normal University having an average publication year of 2021, closely aligned with the current year. Although the citation count is relatively small at 91, this team is likely to emerge as a leading force in future. Regarding author co-citation analysis, 88 authors with at least 73 citations were included (Figure 4B). The top 5 authors with the strongest total link strength (TLS) and citations were Costerton JW, Flemming HC, Donlan RM, Stewart PS, Hoiby N, and Hall-Stoodley L.

**Table 4:** Top 10 active authors in bacterial biofilm eradication field.

Rank	Author	Affiliation	NP	NC	AC	H-index
1	Shunmugiah, Karutha Pandian	Alagappa University	18	959	53.28	14
2	Trampuz, Andrej	Humboldt University of Berlin	18	460	25.56	11
3	Simoes, Manuel	University of Porto	16	634	39.63	10
4	Husain, Fohad Mabood	King Saud University	16	400	25.00	10
5	Hamood, Abdul	Texas Tech University Health Sciences Center	13	150	11.54	5
6	Kim, Young-Mog	Pukyong National University	13	261	20.08	10
7	Tzanov, Tzanko	Polytechnic University of Catalonia	13	328	25.23	7
8	Hancock, Robert E. W.	University of British Columbia	12	1,380	115.00	10
9	Li, Cheng-Hsuan	University of Massachusetts Amherst	12	313	26.08	7
10	Ivanova, Kristina	Polytechnic University of Catalonia	12	301	25.08	6

Ranking: according to the number of total publications. NP, total number of publications; NC, total number of citations; AC, average citations per item.

Meanwhile, a visualization map of co-occurring subject categories is illustrated in Figure 4C generated by Citespace. The top four subject categories ranked by counts were Microbiology, Biotechnology & Applied Microbiology, Pharmacology & Pharmacy, and Biochemistry & Molecular Biology.

### 3.4 Co-cited references, reference burst, and the most-cited publications

**Figure 5:**
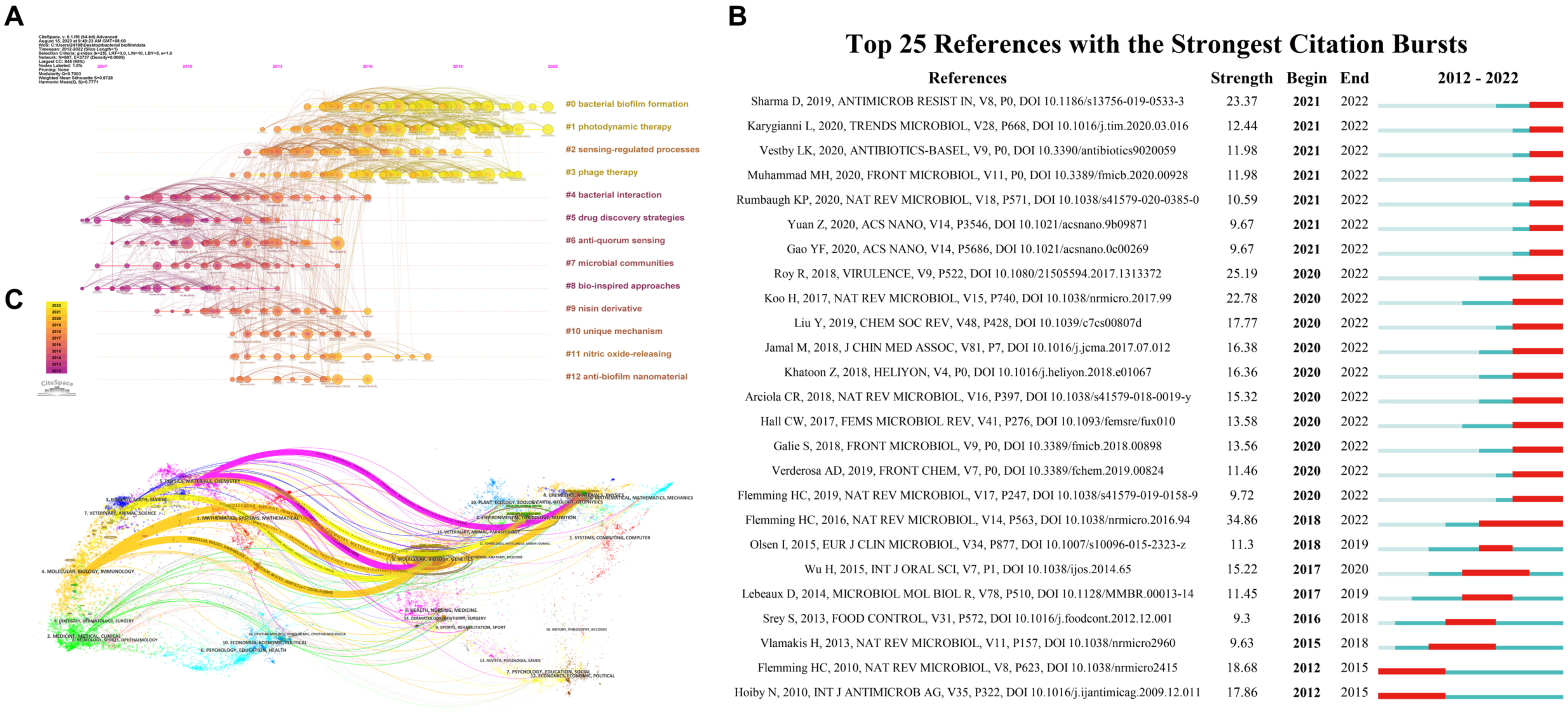
**(A)** Chronological display of co-cited references cluster analysis within the realm of bacterial biofilm eradication. **(B)** The top 25 co-cited references exhibiting pronounced occurrence bursts in the field of bacterial biofilm eradication, as identified by CiteSpace. The timeline is indicated by a blue line, and burst periods are represented using red bars. These bars denote the commencement year, conclusion year, and duration of the burst for each reference. **(C)** A dual map overlay showcasing journals associated with bacterial biofilm eradication according to CiteSpace. The left side displays citing journals, while the right side portrays the cited journals. Citation relationships are illustrated using colored paths, with thicker lines signifying primary citation pathways.

The co-citation analysis of references is presented in Figure 5A conducted by Citespace, where two articles that are cited in another publication are linked through co-citation relationships. Within this chronological perspective, the placement of a node along the horizontal axis signifies its initial emergence, while the interconnecting lines between nodes depict co-citation connections. The dimensions of the nodes are commensurate with the quantity of citations found within the referenced literature. The color spectrum ranging from yellow to purple indicates the relative proximity of the nodes to the years 2022 and 2007, respectively. Furthermore, the timeline view graph of CiteSpace’s reference co-citation analysis provides discernment into the evolution of the field. All included references were categorized into 12 clusters based on their main research themes. It was observed that studies on “bacterial interaction” (Cluster 4), “drug discovery strategies” (Cluster 5), and “bio-inspired approaches” (Cluster 8) were conducted relatively early. On the other hand, current research focuses on areas such as “bacterial biofilm formation” (Cluster 0), “photodynamic therapy” (Cluster 1), and “phage therapy” (Cluster 3). Moreover, citation bursts were examined using the CiteSpace software. The graph of the top 25 citation bursts (Figure 5B) reveals that the first reference with a citation burst appeared in 2012, and the most recent burst of citations occurred in 2021.

The number of citations a paper receives is a measure of its influence and significance. Analyzing highly cited papers can provide perspectives into the research hotspots within the field. In this study, the top 10 most-cited papers were selected based on their average annual number of citations, as presented in Table 5. The most cited article, authored by Hall Clayton W. et al., showing an average of 109.86 citations per year. The second most cited article, with an average of 57.42 citations per year, was published by Seil Justin T. et al. Furthermore, a dual coverage map of journals was created, as depicted in Figure 5C. By analyzing the relationship between the cited literature and the journal in which the cited literature is located, the flow of knowledge information at the journal level is obtained, which intuitively reflects the research trends of the discipline.

**Table 5:** Top 10 highly cited publications in bacterial biofilm eradication field.

Ranking	Title	NC	AC	First author	RF
1	Molecular mechanisms of biofilm-based antibiotic resistance and tolerance in pathogenic bacteria	770	109.86	Hall, Clayton W.	Hall and Mah (2017)
2	Antimicrobial applications of nanotechnology: methods and literature	689	57.42	Seil, Justin T.	Seil and Webster (2012)
3	Strategies for combating bacterial biofilms: A focus on anti-biofilm agents and their mechanisms of action	620	103.33	Roy, Ranita	Roy et al. (2018)
4	Strategies for combating bacterial biofilm infections	538	59.78	Wu, Hong	Wu et al. (2015)
5	Bacterial biofilms: development, dispersal, and therapeutic strategies in the dawn of the post antibiotic era	533	48.45	Kostakioti, Maria	Kostakioti et al. (2013)
6	Bacterial biofilm development as a multicellular adaptation: antibiotic resistance and new therapeutic strategies	505	45.91	de la Fuente-Nunez, Cesar	de la Fuente-Nunez et al. (2013)
7	Biofilms in the food Industry: health aspects and control methods	425	70.83	Galie, Serena	Galie et al. (2018)
8	Quaternary ammonium compounds: an antimicrobial mainstay and platform for innovation to address bacterial resistance	364	40.44	Jennings, Megan C.	Jennings et al. (2015)
9	Molecular mechanisms of antimicrobial tolerance and resistance in bacterial and fungal biofilms	326	32.60	Van Acker, Heleen	Van Acker et al. (2014)
10	Effects of material properties on bacterial adhesion and biofilm formation	318	35.33	Song, F.	Song et al. (2015)

Ranking: according to the number of total publications. NC, total number of citations; AC, average citations per item; PY, publication year; RF, references.

### 3.5 Analysis of keyword co-occurrence and emergent keywords display

**Figure 6:**
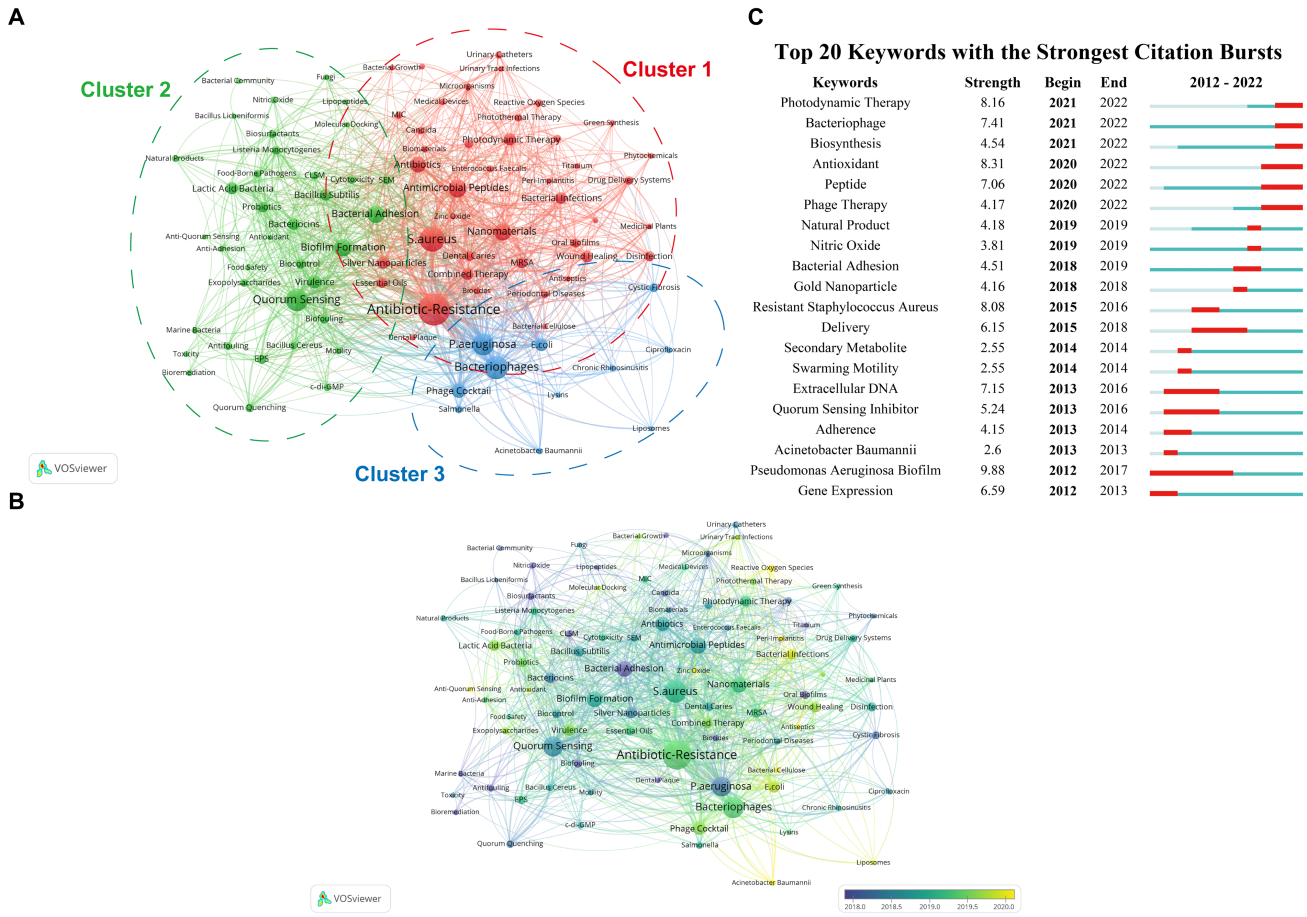
**(A)** A network visualization map illustrating the analysis of keyword co-occurrence. Keywords with close associations are grouped into clusters, each denoted by a distinct color. The keywords are categorized into three clusters: cluster 1 (red nodes), cluster 2 (green nodes), and cluster 3 (blue nodes). **(B)** An overlay visualization map illustrating keyword co-occurrence analysis. Node color corresponds to the average appearing year (AAY) of each keyword. Purple or blue nodes indicate keywords that appeared relatively early in the field, while yellow-coded keywords highlight current research focuses. **(C)** The top 20 keywords exhibiting the most robust citation bursts, as determined by CiteSpace.

In this section, a keyword co-occurrence network map was created using VOSviewer firstly, where closely related keywords were assigned to clusters of the same color. By manually merging keywords with similar meanings and removing irrelevant terms, a total of 90 crucial keywords were identified to represent the article topics. Among them, each keyword should appear no less than 11 times. Furthermore, VOSviewer automatically classified all keywords into several major clusters. As depicted in Figure 6A, the keywords were divided into three categories. Moreover, an overlay visualization map was created to analyze the keyword co-occurrence (Figure 6B), which clearly indicates that the current hot topics are primarily concentrated in Cluster 1 (reactive oxygen species/zinc oxide), Cluster 2 (anti-quorum sensing/*lactic acid bacteria*), and Cluster 3 (*Acinetobacter baumannii*/phage cocktail). The profiles of the top 5 papers with the most citations in three clusters were shown in Table 6.

**Table 6:** Top 5 papers from the first 3 clusters.

Ranking	Title	NC	Profile	RF
#1 No.1	Molecular mechanisms of biofilm-based antibiotic resistance and tolerance in pathogenic bacteria	770	This review summarizes both historical and recent scientific data in support of the known biofilm resistance and tolerance mechanisms.	Hall and Mah (2017)
#1 No.2	Strategies for combating bacterial biofilm infections	538	A review summarized the latest progress in treatment of clinical biofilm infections and scientific investigations, discussed the diagnosis and treatment of different biofilm infections.	Roy et al. (2018)
#1 No.3	Enhanced antibacterial and anti-biofilm activities of silver nanoparticles against Gram-negative and Gram-positive bacteria	316	This research provided proof of the antibacterial and anti-biofilm properties by utilization of *A. cobbe* for producing AgNPs, implying that AgNPs might serve as a supplementary approach in tackling infectious diseases.	Gurunathan et al. (2014)
#1 No.4	Antibacterial effects of silver nanoparticles on gram-negative bacteria: Influence on the growth and biofilms formation, mechanisms of action	315	Antibacterial action of AgNPs on Gram-negative bacteria (planktonic cells and biofilms) is reported in this study.	Radzig et al. (2013)
#1 No.5	Antimicrobial peptides and their therapeutic potential for bacterial skin infections and wounds	231	A review evaluates the potential of AMPs for the treatment of bacterial SSTIs and wounds, providing an overview of the mechanisms of actions of AMPs that contribute to disinfections and wound healing.	Pfalzgraff et al. (2018)
#2 No.1	Effects of material properties on bacterial adhesion and biofilm formation	318	The review summarizes how surface properties influence oral biofilm formation and discusses the important findings from nondental systems that have potential applications in dental medicine.	Song et al. (2015)
#2 No.2	Antibiofilm and quorum sensing inhibitory potential of *Cuminum cyminum* and its secondary metabolite *methyl eugenol* against Gram negative bacterial pathogens	237	Quorum sensing inhibitory (QSI) activity of common South Indian spices and vegetables were evaluated using the bacterial model *Chromobacterium violaceum*.	Sybiya Vasantha Packiavathy et al. (2012)
#2 No.3	Antibiotic discovery: combatting bacterial resistance in cells and in biofilm communities	234	The review compares mechanisms of antibiotic resistance at cellular and community levels based on existing discovery efforts. Future perspectives are also explored.	Penesyan et al. (2015)
#2 No.4	Implication of surface properties, bacterial motility, and hydrodynamic conditions on bacterial surface sensing and their initial adhesion	167	A review about recent works dedicated to understanding the influences of surface charge, surface wettability, roughness, topography, stiffness, and combination of properties on bacterial adhesion.	Zheng et al. (2021)
#2 No.5	Use of potential probiotic *lactic acid bacteria* (LAB) biofilms for the control of *Listeria monocytogenes*, *Salmonella Typhimurium*, and *Escherichia coli O157:H7* biofilms formation	166	The study suggests that the reported potential probiotic strains can be used as alternatives for control of biofilm formation by pathogenic bacteria in the food industry, without conferring a risk to the consumers.	Gomez et al. (2016)
#3 No.1	Biotechnological applications of bacteriophages: state of the art	136	The review discusses the biological nature of bacteriophage particles, their modes of action and potential exploitation in modern biotechnology.	Harada et al. (2018)
#3 No.2	Bacteriophages and their enzymes in biofilm control	120	The article reviews phage based anti-biofilm strategies, emphasizing their ecological aspects of action, with special consideration given to EPS depolymerases.	Chan and Abedon (2015)
#3 No.3	Current trends in development of liposomes for targeting bacterial biofilms	95	A review summarized the latest progress in liposome design for eradicating existing biofilms and preventing biofilm formation, as well as their respective limitations.	Rukavina and Vanic (2016)
#3 No.4	Activity of bacteriophages in removing biofilms of *Pseudomonas aeruginosa* isolates from chronic rhinosinusitis patients	93	The article studied that a single dose of bacteriophage can significantly reduce the biofilm formation of a series of *Pseudomonas aeruginosa* isolates in CRS patients *in vitro*.	Fong et al. (2017)
#3 No.5	Development of a phage cocktail to control proteus mirabilis catheter-associated urinary tract Infections	79	The article reported the isolation of two novel virulent phages, the pod virus VB_PmiP_5460 and the mycovirus VB_PmiM_5461 and its inhibitory effect on the biofilm of *Pseudomonas aeruginosa* was studied.	Melo et al. (2016)

Ranking: according to the number of total publications. NC, total number of citations; RF, references.

We utilized CiteSpace to analyze the top 25 keywords related to outbreaks, as presented in Figure 6C. Within this analysis, we observed keywords that have remained prominent in ongoing outbreaks. These keywords include “*pseudononas aeruginosa biofilm*” (strength = 9.88), “antioxidant” (strength = 8.31), “photodynamic therapy” (strength = 8.16), “*resistant staphylococcus aureus*” (strength = 8.08), and “bacteriophage” (strength = 7.41). These keywords highlight the persistent relevance and focus on these specific topics within the field of outbreak research.

## 4 Discussion

Bacteria forge biofilms as an ingenious facet of their survival strategy, thereby engendering the omnipresence of these intricate structures throughout the natural realm. Stretching back to 1,683, Antoni van Leeuwenhoek observed biofilms for the first time. Nevertheless, the biofilm modus vivendi embraced by microorganisms remained outside the purview of medical microbiologists until the early 1970s (Høiby, 2017). In the continuum of advances, there has been an augmented focus on the intricate mechanics and potential avenues for intervention concerning bacterial biofilm formation. Nonetheless, there persists a notable dearth of comprehensive analyses aimed at synthesizing and prognosticating the trajectory of this burgeoning domain. Therefore, a comprehensive bibliometric analysis of high-impact papers on bacterial biofilm eradication provides meaningful observations into the knowledge structure, current advancements, and research trends in this field.

In this study, we conducted the first bibliometric analysis of articles published between 2012 and 2022, focusing on the history and frontiers of bacterial biofilm eradication. The relevant papers were obtained from the WoSCC database and analyzed using various powerful software tools for constructing and visualizing bibliometric networks and exploring trends and patterns in the areas. These complementary approaches allowed us to gain a systematic understanding of the research landscape in this field.

**Answer for Q1: Based on the information gleaned from published literature, what is the current global dynamic trends of the field?**

In broad terms, the annual counts of publications and citations are the most straightforward indicators for gaging scholars’ research focus in a particular field (Ou et al., 2022). Based on our model, it is projected that the annual publications will reach approximately 671 by the end of 2023. Furthermore, the year 2022 witnessed the highest number of publications (559) and citations (17,938) for papers within the field. The total number of citations for publications amounted to 78,801, with expectations of reaching another peak in 2023 and continuing to rise. Our analysis revealed a notable increase in the annual number of publications and total citations over the past 11 years. These statistics highlight the considerable focus and interest that research in this field has attracted (Pouliliou et al., 2020).

**Answer for Q2: In this field, which countries/regions, institutions, journals, and authors have demonstrated the highest levels of productivity and influence?**

Tracking the research contributions of countries, institutions, and teams offers invaluable perspectives into the current delve tendency within the field. Among all countries, the USA and China emerged as the leading contributors with 626 and 597 papers published, respectively. The higher number of publications suggests a greater social demand for research compared to others. However, findings indicate that the USA exhibits greater influence and innovation and positioned as a pioneer. China should focus on expanding international accessibility from universities and institutions to enrich its strategies and enhance its research impact. In response to the wave of globalization, collaborations between countries have become increasingly common. The high impact of the USA may be attributed, in part, to its effective collaboration with other countries. However, some countries lack sufficient collaboration with their counterparts. In consequence, promoting collaborations with these countries could be an approach to deepen research and applications in this domain. Collaboration fosters knowledge exchange, innovative ideas, and the pooling of resources, could ultimately benefit the field.

In terms of the most productive institutions, the institute of the CAS ranked first in terms of the number of articles published, followed by the Egyptian Knowledge Bank and King Saud University (Figure 3A; Table 2). Regarding the average count of citations per article, CAS was 32.1 per article, while the first place, Aligarh Muslim University in India, was 44.94 per article. This is in line with the findings of the national-level studies. Therefore, national research should enhance the depth of research and accessibility of papers, thereby increasing their impact. The results also show that top domestic and international institutions are always an important force. Besides, 40 % of the top 10 most active funding bodies are from China and the USA (Figure 3B). There is insufficient international collaboration between institutions from different countries, with most collaborations occurring among domestic institutions. Obviously, cooperation between institutions in different countries are not close enough and that most countries just cooperated within their own countries.

Furthermore, the journal “Frontiers in Microbiology” were the most influential source according to publications and co-citation analysis, far ahead of the second place “Antibiotics Basel” (Figure 3C; Table 3). Moreover, Pandian Shunmugiah Karutha, from Alagappa University, was the most published and cited author with 18 papers and a total of 959 citations, while Deng Le and Yang Ke from Hunan Normal University is likely to emerge as a leading force in future (Table 4). Besides, the most cited paper addressed a comprehensive review amalgamates historical and contemporary scientific insights that validate the recognized resistance mechanisms inherent in biofilms and raised some suggestions about prospective avenues for further research (Hall and Mah, 2017). The second paper encompassed a review of pertinent literature in antimicrobial applications of nanotechnology and conducted a concise overview of bacteriostatic and bactericidal mechanisms (Seil and Webster, 2012). The third one undertook the task of aggregating all established approaches or focal points aimed at countering biofilm formation, standing to provide invaluable guidance for researchers in crafting novel compounds endowed with anti-biofilm properties (Roy et al., 2018). Regarding reference co-citation analysis, the heated topic were bacterial biofilm formation, photodynamic therapy, and phage therapy. Correspondingly, Hall, Clayton W possesses the highest citations per year (109.86), indicating the significant impact of this paper (Table 5). In summary, these highly cited directions are also comprehensive compasses for the forefront and hotspots of disciplinary development.

**Answer for Q3: What are the primary areas of research focal points and the emerging topics of intense interest for upcoming research?**

Research focal points could be displayed by utilizing keywords and references analysis, facilitating the exploration of cutting-edge research frontiers and emerging trends. As depicted in Figure 5A, references were mainly categorized into 12 clusters, compared with the earlier directions including “bacterial interaction,” “drug discovery strategies,” and “bio-inspired approaches,” recent investigations tended to fix eyes on directions like “bacterial biofilm formation,” “photodynamic therapy,” and “phage therapy.” In addition, a visualization map of co-occurring subject categories was illustrated in Figure 4C, and the top four subject categories were Microbiology, Biotechnology & Applied Microbiology, Pharmacology & Pharmacy, and Biochemistry & Molecular Biology. As illustrated in subsequent analysis (Figure 5C), the intersection of journals in the biplot indicates that articles published in journals related to molecular biology, genetics, chemistry, materials, physics, environmental science, toxicology, and nutrition are primarily referenced by papers appearing in fields including molecular biology, immunology, physics, materials, chemistry, and veterinary and animal sciences. Although there are citation connections from medical and clinical sources as well, they have not yet established a prominent pathway, this concerted endeavor is crucial in propelling the shift from fundamental research toward tangible applications in this field.

Moreover, references citation bursts indicate articles that have experienced a significant increase in citations within a certain period, suggesting that the content covered in these articles was quickly recognized and disseminated within the research field. The top 25 citation bursts (Figure 5B) reveal that the first reference with a citation burst began in 2012, and the most recent burst of citations appeared in 2021. In parallel, conducting keyword analysis can further identify emerging topics and predict future research prospects and hotspots in the field. In the present study, a total of 90 crucial keywords were screened and classified into three major clusters. As displayed in Figure 6A, the keywords were divided into three categories: Cluster 1 (red nodes, focus on antibiotic-resistance/nanomaterials/photodynamic therapy), Cluster 2 (green nodes, mainly on quorum sensing/biofilm formation/*lactic acid bacteria*), and Cluster 3 (blue nodes, fixes on bacteriophages/phage cocktail). Furthermore, Figure 6B illustrates that the prevailing subjects of interest are predominantly clustered within reactive oxygen species/zinc oxide, anti-quorum sensing/*lactic acid bacteria*, and *Acinetobacter baumannii*/phage cocktail. To delve into outbreaks, we further analyzed the leading 25 keywords, as illustrated in Figure 6C, these keywords mainly encompass “antioxidant,” “photodynamic therapy,” and “bacteriophage,” underscoring the enduring relevance and concentrated focus on these specific subjects within the realm of outbreak research, and are also almost consistent with results of citation bursts analysis part. The profiles of the most highly cited papers within these three clusters are detailed in Table 6. By examining the contents and viewpoints presented in these specific documents, we can attain the most exhaustive overview and pioneering analysis within the domain.

As is known to all, the comprehensive exploration of intricate mechanisms and pivotal molecular cues intrinsic to the process of biofilm formation not only furnishes a theoretical framework but also furnishes propitious instruments to foster the evolution of finely tailored strategies for biofilm eradication. In brief, biofilm-associated existence triggers discernible phenotypic alterations in the bacterial entities, characterized by a downregulation in the transcriptional gene expression and translational activity of proteins that are pivotal for bacterial cellular metabolism. The culmination of these changes results in an evident decrease in metabolic vitality (Liu et al., 2019; Sharma et al., 2019). The process of establishing a biofilm augments the expression profiles of pertinent genes, encompassing factors such as adhesion, quorum sensing mechanisms, and competence (Karygianni et al., 2020). Following successful adhesion, the secretion of the extracellular matrix (ECM) layer ensues, signifying the initiation of biofilm formation and its subsequent maturation phase. Under specific conditions, the biofilm disbands, facilitating its expansion and colonization in other sites (Solano et al., 2014; Hengge, 2019; Karygianni et al., 2020). Governing these intricate dynamics is a regulatory system recognized as quorum sensing (QS), where signaling molecules amass proportionately with cellular density (Solano et al., 2014). These molecules wield a pivotal role in nurturing the maturation and dispersion of biofilms, concurrently regulating the expression of genes and proteins that are integral to biofilm development. Our investigative findings further reveal a substantial body of research dedicated to delving into diverse facets such as biofilm formation, quorum sensing, quorum quenching, c-di-GMP signaling, EPS matrix composition, and bacterial adhesion phenomena, providing more beneficial assistance for the application of anti-biofilm strategies. Indeed, current and emerging anti-biofilm strategies are deeply cross-fertilized from “mechanism” to “strategy” in terms of disrupting QS, preventing bacterial adherence to surfaces, stopping bacterial aggregation in the viscous mucus layer, degrading EPS, and developing nanoparticle-based anti-microbial drug complexes targeting per sisters in the core of biofilms, but they still face practical barriers to their application, such as limited *in vivo* efficacy, potential cytotoxicity to the host cell and the tendency to induce drug resistance in the targeting of biofilm-forming micro-controllers (Nett et al., 2016; Howlin et al., 2017; Louis et al., 2022).

Moreover, *Staphylococcus aureus*, *Pseudomonas aeruginosa*, *Acinetobacter baumannii*, and *Escherichia coli* stand out as acknowledged pathogens within the realm of medical environments. Their capacity to manifest a spectrum of virulence factors, prominently exemplified by biofilm formation, assumes a pivotal role in the pathogenesis they underpin (Gedefie et al., 2021; Sedarat and Taylor-Robinson, 2022). Our investigational insights further underscore the predominant focus of research on the eradication of biofilms instigated by these bacterial agents. Of noteworthy significance is the recent surge of attention directed toward the meticulous exploration of *Acinetobacter baumannii* biofilms, marking an unmistakable trend that is concurrently emerging and prevailing. *Acinetobacter baumannii* occupies a unique taxonomic niche, and its clinical significance resonates throughout literature, accentuated by the escalating prevalence of carbapenem-resistant strains across healthcare settings and communities alike (Morris et al., 2019). In response to this critically exigent pathogenic challenge, the exploration of combination therapies stands poised as an innovative avenue, potentially affording a novel approach to mitigation (Srisakul et al., 2022).

Based on the meticulous and in-depth professional research coupled with extensive interdisciplinary integration, a continuum of efficacious strategies to counter biofilm formation has been consistently and progressively put forth. As revealed in our results, diligent researchers have achieved notable progress across diverse fronts of bacterial biofilm eradication. These encompass realms including natural product interventions (Shamim et al., 2023), antimicrobial peptide regimens (Scheper et al., 2021), probiotic antagonistic strategies (Gomez et al., 2016), gas-mediated treatments (Koo et al., 2017), bacteriophage-based therapies (Akbarian et al., 2022), sophisticated drug delivery employing nanomaterials (Choi et al., 2023), photodynamic therapy harnessing reactive oxygen species, photothermal treatments, and synergistic combination therapies (Varma et al., 2023), etc. These intricate and multidimensional endeavors have played a pivotal role in propelling the historical trajectory of bacterial biofilm elimination to new heights.

In general, these various approaches for eliminating biofilms offer promising avenues for mitigating biofilm-related risks. However, the persistence of infections linked to biofilms remains a significant public health concern. Thus, gaining further insights into the mechanisms governing the formation of biofilms facilitates the utilization of agents targeting microbial components. Despite recent progress, both the current and proposed anti-biofilm agents, along with their *in vivo* applications, necessitate thorough research to ensure their efficacy and safety in clinical contexts. Additionally, certain recent technological strides, such as enzyme engineering and DNA-based sequencing, exhibit substantial potential for more potent anti-biofilm tactics (Kilic and Bali, 2023). Achieving the transition of multiple anti-biofilm treatments from lab settings or controlled environments to real-world clinical practice demands collaborative efforts across various fields. Biomedical researchers, microbiologists, chemists, and engineers all play pivotal roles in advancing this critical endeavor. Hence, this bibliometric analysis serves as a guiding framework to further unify endeavors in delving into the genetics, physiology, and dynamics of bacterial biofilms, as well as the mechanisms driving their antimicrobial resistance. It aims to foster deeper integration of communication at national, institutional, team, and funding levels, and to stay abreast of research focal points and emerging topics that could catalyze the advancement of current and emerging technologies for biofilm eradication strategies. Together, we aspire to make significant strides in surmounting the existing barriers in transitioning anti-biofilm strategies from the “fundamental” to the “practical” realm.

## 5 Limitations

This study has several limitations that should be acknowledged. Firstly, the data utilized in this analysis were solely obtained from WoSCC, which although comprehensive, may not encompass all relevant publications. Additionally, the varying quality of articles included in this study introduces potential biases and undermines the overall credibility of the analysis. Moreover, it is important to recognize that no matter what bibliometrics tool does have inherent limitations. When extracting terms from titles, abstracts, and keywords in literature, the process of cluster analysis can display notable fluctuations, and there is no assurance that all terms conveying similar meanings will be accurately amalgamated. It is desirable for future studies to explore multiple databases to obtain a comprehensive understanding of the worldwide bacterial biofilm eradication investigations. This will help in gaining a more complete and holistic perspective on the topic.

## 6 Conclusion

To the best of our knowledge, this study represents the first comprehensive bibliometric analysis of bacterial biofilm eradication research, illustrating its current research status and global dynamic trends. The findings indicate that this field is rapidly advancing and is expected to facilitate greater expansion in the times ahead. China and the USA emerge as the primary driving forces and occupy a central position in global research on the topic. To foster progress, future researchers should prioritize enhancing collaboration among different countries/regions. The institute of the CAS and the journal of “Frontiers in Microbiology” exhibit the highest levels of productivity and influence. Pandian Shunmugiah Karutha was the most published and cited author while Deng Le with his team is poised to become a dominant influence in the future.

Notably, “bacterial biofilm formation”, “photodynamic therapy” and “phage therapy” are currently regarded as research hotspots. Looking for future, promising research directions may focus on achieving the transition of numerous biofilm eradication strategies from controlled laboratory environments or animal studies to practical clinical settings through a deepen collaborative approach across various disciplines.

In essence, this bibliometric research offers a thorough examination of research on biofilm elimination, presenting valuable sources of information for researchers and decision-makers and playing a guidelines role in advancing the transition from foundational research to practical applications in this field.

## Author contributions

TW: Data curation, Formal analysis, Methodology, Software, Visualization, Writing – original draft. RZ: Conceptualization, Methodology, Supervision, Validation, Writing – original draft, Writing – review & editing. ZC: Data curation, Formal analysis, Methodology, Software, Visualization, Writing – original draft. PC: Data curation, Software, Validation, Writing – original draft. QZ: Data curation, Software, Visualization, Writing – original draft. QW: Funding acquisition, Project administration, Supervision, Writing – review & editing, Resources, Validation.

## Footnotes

^1^ https://www.scimagojr.com/
